# Insensitivity to the Spatial Repellent Action of Transfluthrin in *Aedes aegypti*: A Heritable Trait Associated with Decreased Insecticide Susceptibility

**DOI:** 10.1371/journal.pntd.0003726

**Published:** 2015-04-16

**Authors:** Joseph M. Wagman, Nicole L. Achee, John P. Grieco

**Affiliations:** 1 Department of Preventive Medicine and Biometrics, Division of Tropical Public Health, Uniformed Services University of the Health Sciences, Bethesda, Maryland, United States of America; 2 College of Biological Sciences, Eck Institute for Global Health, University of Notre Dame, Notre Dame, Indiana, United States of America; Centers for Disease Control and Prevention, UNITED STATES

## Abstract

**Background:**

New vector control paradigms expanding the use of spatial repellents are promising, but there are many gaps in our knowledge about how repellents work and how their long-term use might affect vector populations over time. Reported here are findings from a series of *in vitro* studies that investigated the plasticity and heritability of spatial repellent (SR) behaviors in *Aedes aegypti* exposed to airborne transfluthrin, including results that indicate a possible link between repellent insensitivity and insecticide resistance.

**Methodology/principal findings:**

A dual-choice chamber system was used to observe directional flight behaviors in *Aedes aegypti* mosquitoes exposed to passively emanating transfluthrin vapors (1.35 mg/m^3^). Individual SR responder and SR non-responder mosquitoes were identified, collected and maintained separately according to their observed phenotype. Subsequent testing included re-evaluation of behavioral responses in some mosquito cohorts as well as testing the progeny of selectively bred responder and non-responder mosquito strains through nine generations. At baseline (F_0_ generation), transfluthrin actively repelled mosquitoes in the assay system. F_0_ mosquitoes repelled upon initial exposure to transfluthrin vapors were no more likely to be repelled again by subsequent exposure 24h later, but repelled mosquitoes allowed to rest for 48h were subsequently repelled at a higher proportion than was observed at baseline. Selective breeding of SR responders for nine generations did not change the proportion of mosquitoes repelled in any generation. However, selective breeding of SR non-responders did produce, after four generations, a strain of mosquitoes that was insensitive to the SR activity of transfluthrin. Compared to the SR responder strain, the SR insensitive strain also demonstrated decreased susceptibility to transfluthrin toxicity in CDC bottle bioassays and a higher frequency of the V1016I^*kdr*^ mutation.

**Conclusions/significance:**

SR responses to volatile transfluthrin are complex behaviors with multiple determinants in *Ae*. *aegypti*. Results indicate a role for neurotoxic irritation of mosquitoes by sub-lethal doses of airborne chemical as a mechanism by which transfluthrin can produce SR behaviors in mosquitoes. Accordingly, how prolonged exposure to sub-lethal doses of volatile pyrethroids might impact insecticide resistance in natural vector populations, and how already resistant populations might respond to a given repellent in the field, are important considerations that warrant further monitoring and study. Results also highlight the critical need to develop new repellent active ingredients with novel mechanisms of action.

## Author Summary

There is growing evidence to support the expanded use of spatial repellents for vector control, but there are still many uncertainties about how repellents work and how their long term use may impact vector populations over time. Here, we conducted a series of *in vitro* experiments that investigated spatial repellent (SR) behaviors in *Aedes aegypti* mosquitoes exposed to airborne transfluthrin, a volatile pyrethroid commonly used in repellent products. We were able to show that repellent insensitivity is linked to reduced insecticide susceptibility and increased knock down resistance allele frequency, indicating that sub-lethal doses of airborne transfluthrin can elicit SR behaviors in mosquitoes by inducing an agitated state via neurotoxic pathways independent of olfactory stimulation. This raises questions about how the use of volatile pyrethroid repellents may impact insecticide resistance in target vectors over time, highlighting the need to further understand all of the physiological drivers of SR behaviors and emphasizing the requirement to develop new repellent active ingredients with novel, non-toxic mechanisms of action.

## Introduction

New vector control tools and paradigms are desperately needed to complement existing approaches [1–3], and there is growing evidence to support the expanded use of spatial repellents to help address this need [4–9]. The ultimate goal of public health interventions utilizing repellents is to exploit the behavior modifying effects of certain chemicals to prevent human-vector contact and, therefore, reduce disease transmission. Such approaches are among the most promising new strategies under investigation, with much progress already shown towards defining the parameters of spatial repellent-based interventions to control the global arbovirus vector *Ae*. *aegypti* [10–13]. However, there are gaps in our knowledge about how repellents work, including the exact molecular and physiological mechanisms by which various chemicals elicit SR behaviors in important vector species [5, 14–17] and the hereditary basis by which SR behavioral traits are maintained in populations of disease vectors [18, 19].

Spatial repellency (SR) is one of several behavior modifying effects of insecticides on mosquitoes that have been recognized for decades [6, 8] and have been shown to contribute to disease reduction in many settings [5, 20, 13]. In outlining a new classification system to more accurately describe the actions of chemicals used for malaria vector control, Grieco et al. (2007) defined SR actions as those that stimulate “movement away from the chemical source without the mosquito making physical contact with the treated surface” [6]. An expanded concept of SR, which also includes chemical actions that interfere with host detection and/or otherwise disrupt the blood-feeding process, was established by WHO in 2013 to help determine guidelines for efficacy testing [9]. Taken together, it is clear that what is casually referred to as spatial repellency is really a set of complex and multifactorial behaviors which can be generally thought of as reactions to air-borne chemical stimuli that deter mosquitoes from entering a space to take a blood meal from an otherwise suitable host.

Despite the complexities inherent in the modification of mosquito behavior, much evidence to date seems to indicate that olfactory mechanisms underlie many repellent behaviors [17, 21, 22]. For example, DEET, which is probably the most widely used and thoroughly studied mosquito repellent [23, 24], is thought to work either through direct olfactory stimulation [16, 25] and/or through interference with normal host cue detection, essentially masking the presence of a potential blood meal [14, 26]. Although DEET is typically found in products labeled for personal protection that are applied directly to the skin and is not, strictly speaking, a spatial repellent able to protect occupants of a defined area, knowledge of its mechanisms of action is likely to inform much of our view of how SR compounds function. Indeed, epidemiological and entomological evidence garnered from the use of indoor residual spraying with DDT for malaria control also supports a model whereby the SR action of the chemical results from a separate mechanism, likely olfaction, from that which produces neurotoxicity: SR activity is preserved in many locations where insecticide resistance is widely reported [27]. Similar observations have also been reported in pyrethroid tolerant mosquitoes that still demonstrate behavioral avoidance to sub-lethal doses of various pyrethroids [28, 29, 15]. Additionally, it has also long been observed that some proportion of mosquitoes continue to locate hosts and feed even in the presence of a repellent [30, 31], and in *Ae*. *aegypti* this DEET insensitivity has been shown to be a heritable trait with incomplete penetrance [19] associated with specific odorant receptor polymorphisms [32, 26].

Less clear, however, is whether or not olfactory pathways are the only physiological drivers of SR behaviors in mosquitoes. For instance, Ogoma et al. (2014) have reported that airborne pyrethroids and DDT both elicit multiple behavioral effects on a given mosquito population at the same time, *including* deterrence (the prevention of mosquito entrance into a structure), irritancy and excito-repellency (eliciting the premature exit of mosquitoes from a structure via physical contact with an insecticide treated surface or with insecticide vapors, respectively), reduced blood feeding, increased 24h mortality and reduced fecundity [7]. Kawada et al. recently reported reduced pyrethroid (permethrin and deltamethrin) contact repellency in a strain of *Anopheles gambiae* s.s. with the L1014S^*kdr*^ mutation, but not in strains of *An*. *arabiensis* or *An*. *funestus* s.s. with cytochrome P450 driven metabolic resistance traits, supporting a role for the non-lethal disruption of neuronal sodium ion channel function in eliciting the observed excito-repellency/irritancy behaviors [15]. While they did not evaluate SR behaviors specifically, these results are in line with previous knowledge that many pyrethroid compounds (i.e., permethrin, deltamethrin and alphacypermethrin) can induce irritant and/or hyperactive responses in mosquitoes at sub-lethal concentrations [33, 34] and this hyperactivity can promote the avoidance of insecticide treated nets [35]. It is clear that physical contact with surfaces treated with these pyrethroid insecticides can produce repellency behaviors through neurologically disruptive mechanisms. It is unknown, however, whether or not a highly active and more volatile pyrethroid insecticide like transfluthrin, which also has SR properties [36, 37, 12, 7], elicits the same physiological responses through airborne exposure. This question is especially important as residual pyrethroids are currently the most commonly used class of public health insecticide worldwide and there are growing concerns about the rapid expansion of pyrethroid resistance in key vector species [5, 38, 39]. Critically, it is unclear how the use of volatile compounds that could act through the same physiological pathways as the most commonly used residual insecticides might complicate the insecticide resistance landscape.

Given the complex and multifactorial nature of SR behaviors in mosquitoes, the molecular and hereditary drivers of the behavior are likely to vary across different active ingredients and target organisms. Nonetheless, elucidating which mechanisms dominate in specific transmission settings is an important step to understanding how to best use spatial repellents in a public health context [40] and how their long-term use might impact vector populations over time [6, 29]. Additionally, this data could be used to guide the rational design of new active ingredients that mitigate resistance driving mechanisms [5]. Here, we report on a series of *in vitro* experiments that first examined the plasticity and heritability of non-contact SR behaviors in *Ae*. *aegypti* that were exposed to airborne transfluthrin, and subsequently explored a link between SR insensitivity and reduced insecticide susceptibility in a selectively bred strain of this important arbovirus vector.

## Methods

### Test mosquitoes

*Aedes aegypti* (L.) mosquitoes were colonized from wild-caught (P_1_) larvae collected from discarded automobile tires near the Belize Vector and Ecology Center (BVEC) in Orange Walk Town, Belize (18°04.938’N, 88°33.390’W). The P_1_—F_4_ generations were reared and tested at the BVEC field laboratory at ambient light, temperature and humidity. Later generations (F_5—_F_10_) and experimental crosses were reared and tested under climate controlled conditions (28°C, 60% RH, and 12L:12D light-dark schedule) at the Uniformed Serviced University of the Health Sciences (USUHS) in Bethesda, MD. Larvae were fed Chiclid Gold fish pellets (Kyorin Co., LTD, Himeji, Japan) and adults were provided 10% sucrose solution from soaked cotton *ad libitum*. Using CDC bottle bioassay methods, F_0_ adults exhibited greater than 90% susceptibility to transfluthrin, malathion and DDT at 60 minutes (S1 Fig). SR Behavioral assays were performed using 5–12 day old mosquitoes, which were sorted into cohorts of 20 mosquitoes approximately 24h prior to testing. Female test mosquitoes were unmated, to allow for downstream selective breeding, and were sugar starved (provided only water-soaked cotton) for approximately 24h before testing, following standardized methods [41]. Because high mortality rates were observed in male mosquito populations, they were not sugar starved prior to testing.

### SR behavioral bioassay

**Fig 1:**
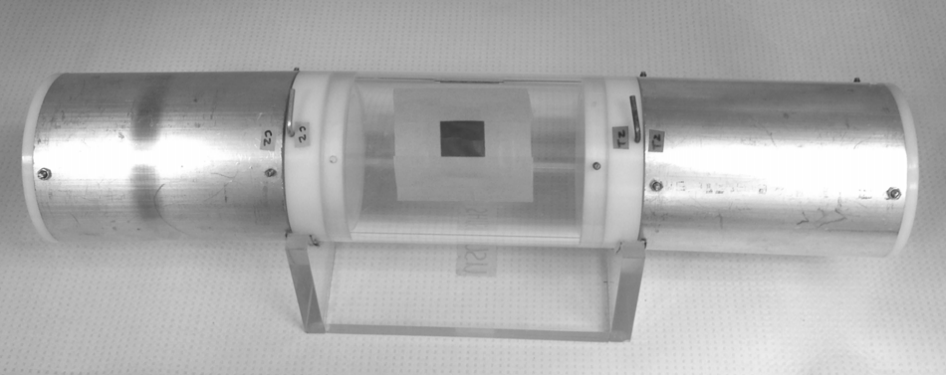
The high throughput screening system (HITSS) spatial repellency assay (SRA). The treatment chamber (right hand metal cylinder) is covered internally by nylon organdy netting treated with transfluthrin dissolved in 100% acetone. The control chamber (left hand metal cylinder) contains netting treated with acetone only. Cohorts of 20 mosquitoes are introduced into the central (clear) chamber and directional flight behaviors are observed (See text, Adapted from Grieco, et al. 2007. *J Am Mosq Control Assoc* 2005, 21:404–411).

SR behavior was evaluated using a high throughput screening system (HITSS-SRA configuration) (Fig 1), previously described by Grieco et al. (2007) [41] and recently adopted by the WHO as a standard procedure for *in vitro* efficacy testing of spatial repellents [9]. The dual-choice chamber system, which allows the observation of directional mosquito movement in response to a single chemical stimulus outside the context of host cues, consists of a clear Plexiglas central unit connected at opposite ends to one treatment chamber housing repellent-treated netting and one control chamber housing a net treated with acetone only (Fig 1). Tests were conducted to evaluate *Ae*. *aegypti* SR responses to passively emanating transfluthrin (2,3,5,6-tetrafluorobenzyl (1R)-trans-3-(2,2-dichlorovinyl)-2,2-dimethyl cyclopropanecarboxylate) (S.C. Johnson and Son, Inc., Racine WI), a volatile synthetic pyrethroid with widely demonstrated SR efficacy against mosquitoes [7, 36, 37, 12]. Briefly, reagent grade (unformulated) transfluthrin was dissolved in 100% acetone (Hofius Ltd./Ace Hardware, Belize City and Fisher Scientific, Waltham MA). This solution was then applied evenly by micropipette across the surface of 11cm x 25cm pieces of nylon organdy netting (No. I10N, G-Street Fabrics, Bethesda MD) and allowed to air dry a minimum of 15 minutes before use. Industry guidelines (M.C. Meier, personal communication, 16 August 2011) and concurrent experimental hut studies using transfluthrin in Belize [42] indicate a standard field application rate (FAR) of 1.35mg active ingredient per cubic meter of airspace to produce indoor SR activity against mosquitoes via passive emanation. Accordingly, HITSS treatment nets delivering 1x the FAR into the assay system were treated with 0.9mL of a 2.2x10^-6^ M (8.4x10^-4^ mg/mL) solution. Concentrations tested ranged from 0.5xFAR to 1000xFAR. Control nets were treated with 100% acetone only.

### Test procedure and spatial activity index

Cohorts of 20 mosquitoes were introduced into the central HITSS chamber and, after a 30 second acclimation period, butterfly valves situated at both ends of the central chamber were opened simultaneously to allow free movement of mosquitoes in either direction into either end chamber. After a ten minute exposure period, the butterfly valves were closed and the numbers of mosquitoes in each chamber were counted. Spatial repellency is measured by considering the number of mosquitoes that have moved into the untreated, control chamber (away from the treated surface) relative to the total number of mosquitoes that have moved in either direction using a weighted spatial activity index (SAI), equal to [(N_c_- N_t_)/(N_c_+ N_t_)]x[(N_c_+ N_t_)]/N] where N is the total number of mosquitoes per replicate and N_c_ and N_t_ are the number of mosquitoes in the control and treatment chambers, respectively. Possible values for the weighted SAI range from 1 to -1, with a value of 1 indicating the strongest SR response possible (movement of all mosquitoes away from the chemical source), zero indicating no net response, and a value of -1 indicative of a strong attractive response (movement of all mosquitoes towards the chemical source). To account for mosquito mortality, the total number of mosquitoes tested per each replicate was corrected using Abbott’s formula [43].

### HITSS SR dose-response curve

A SR dose-response curve was established using unselected (control) females by varying the dose of transfluthrin in the HITSS treatment chamber and measuring differences in corresponding SAI values and overall assay mortality (S2 Fig). The dose corresponding to 1xFAR (1.35 mg/m^3^) produced the largest SAI value (0.10, significantly greater than zero at *P*<0.02) and an overall non-contact mortality of only 2.8% and was selected for use in all subsequent HITSS SR replicates.

### General approach

Male and unmated nulliparous female mosquitoes were tested separately and, after each experimental replicate, were identified as either SRA responders (SRA^+^) if they had escaped into the untreated control chamber or SRA non-responders (SRA^-^) if they either stayed in the central chamber or flew into the treatment chamber (S3 Fig). Mosquitoes that were located in the treatment chamber at the end of a replicate (i.e. had made physical contact with the transfluthrin treated netting) were enumerated for statistical purposes but then discarded and not further processed or analyzed. Though both male and female mosquitoes were tested during these experiments, only female behavior was analyzed statistically and only female results are presented here. Typically, males were tested in fewer replicates only to provide sufficient numbers of each behavioral phenotype (SRA^+^ responders and SRA^-^ non-responders) for selective mating purposes.

### Behavioral plasticity

To evaluate the plasticity of SR responses in unselected F_0_ females exposed to transfluthrin, test replicates were performed and mosquitoes were immediately collected and maintained separately based on their observed behavioral phenotype, i.e. SRA^+^ responders and SRA^-^ non-responders. Mosquitoes were re-assayed on a subsequent day (day 2), after either a 24h or 48h resting period, and the weighted SAI for each phenotype cohort was compared to baseline (day 1) results using Student’s t-test at 95% confidence.

### Heritability of SR behaviors

The heritability of SR behavioral responses was evaluated by performing test replicates and collecting mosquitoes based on their SR behavioral phenotype, as described above (S3 Fig). SR responder females were then selectively mated with SR responder males to establish an SRA^+^ strain of *Ae*. *aegypti*, and non-responder females were mated with non-responder males to establish an SRA^-^ strain. Changes in the SAI scores in test populations from each strain were followed for 9 generations and were compared using ANOVA with Dunnett’s test for multiple comparisons at 95% confidence. An additional control strain of *Ae*. *aegypti* originating from the same field collected P_1_ larvae but which was allowed to freely mate was also maintained and tested.

### Insecticide susceptibility testing

In order to monitor relative changes in transfluthrin insecticide susceptibility over time and across different experimental populations, CDC bottle bioassay tests [43] were performed at various selection points, including the F_0_, F_5_ and F_8_ generations and in progeny from an experimental cross between F_9_ SRA^-^ females and newly colonized wild type F_0_ males. A discriminating dose of 94 ng transfluthrin (0.25 nm, approximately 0.125xFAR) per bottle was established using F_2_ unselected control females (S1 Table). Test replicates lasted one hour, with mosquito knockdown recorded every 15m and final mortality recorded at 24hr.

### *kdr* allele frequencies

Using the PCR genotyping approach developed by Linss et al. (2014) [44], *Ae*. *aegypti* voltage gated sodium ion channel V1016I and F1534C *kdr* allele frequencies were estimated using cohorts of 30 mosquitoes each from the F_9_ Control, SRA^+^ and SRA^-^ populations and the experimental cross progeny. Both target site mutations have been previously observed in *Ae*. *aegypti* populations from Latin America and the Caribbean and have been shown to contribute to pyrethroid resistance [45, 46, 44].

### Statistical analysis

Unless otherwise noted, SAI scores were calculated for each test population at each time point using 180 total mosquitoes, consisting of 9 replicates of 20 mosquitoes each, following established procedures [9]. Herein, the term ‘test population’ is used to refer to a sample of mosquitoes from a unique generation (e.g. F_3_) of a unique behavioral phenotype ‘strain’ (e.g. SRA^-^, SRA^+^ or control). Raw data was organized and descriptive analyses were performed using Excel 2007 (Microsoft Corp., Albuquerque NM). A non-parametric signed rank test (PROC UNIVARIATE) in SAS v8 statistical software (SAS Institute Inc., Cary, NC) was used to determine if mean SAI values were different from zero for each test population. SAI values were compared between populations via Student’s t-test and ANOVA with Dunnett’s test for multiple comparisons using SPSS Statistics 22 software (IBM Corp., Armonk NY). The *kdr* allele frequencies and herterozygosity were compared using Z-tests on the difference between sample proportions, and a chi-square test with one degree of freedom was used to evaluate deviations from Hardy-Weinberg equilibrium [47]. All analyses were performed at α = 0.05.

## Results

### Behavioral plasticity

**Fig 2:**
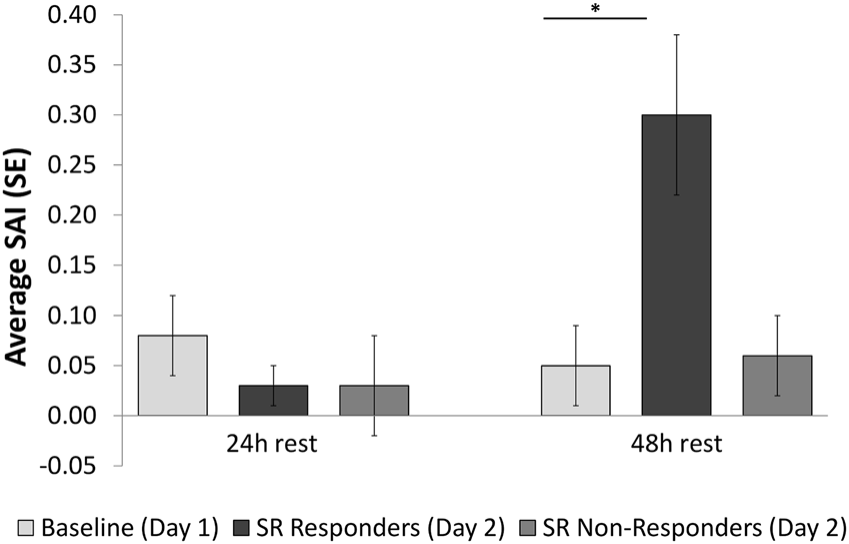
Plasticity of spatial repellency behaviors. Weighted spatial activity index (SAI) scores for cohorts of *Aedes aegypti* females exposed to 1.35mg/m^3^ transfluthrin. After observing baseline (Day 1) behaviors, test mosquitoes were re-assayed on a subsequent day following either 24 or 48 hours of resting. * indicates a day 2 SAI significantly different than the baseline day 1 SAI, *P*<0.05.

Two variations of the behavioral plasticity experiment were performed using F_0_ mosquitoes, with differing results (Table 1 and Fig 2). During the first experiment, mosquito cohorts (total n = 180 mosquitoes, average baseline SAI = 0.08 ±0.03 SEM) were re-assayed after a 24 hour rest period and results indicated a large degree of plasticity in behavioral responses to the repellent: mosquitoes repelled on day one (n = 29) were not more likely to be repelled again on day two (SAI = 0.03 ± 0.02) (Fig 2). Mosquitoes not repelled on day one (n = 129) were equally unlikely to be repelled on day two

(SAI = 0.03 ±0.04) (Fig 2). For the second experiment, mosquitoes (total n = 280, average baseline SAI = 0.05 ±0.04) were not re-assayed until the second day after the original test (48 hours post exposure). Unlike mosquitoes that were allowed to rest for 24hr, day one repellent responders from this cohort (n = 60) were more likely to be repelled again on day two (SAI = 0.30 ±0.08, *P*<0.05) (Fig 2). As was observed in the first experiment, non-responding mosquitoes from this experiment (n = 155) were also equally non-responsive on day two (SAI = 0.06±0.04) (Fig 2).

**Table 1:** Plasticity of spatial repellency behaviors in Aedes aegypti^1^ females exposed to volatile transfluthrin (1.35 mg/m3).

Rest Period	Cohort	Number of Trials (No. Mosqu.)	Mean Percent Active (SEM)	Mean SAI^2^ (SEM)	SR^3^	*P* ^4^
24h	Baseline (Day 1)	9 (180)	24 (19)	0.08 (0.03)	25	0.04
	Responders (Day 2)	2 (29)	17 (4)	0.03 (0.02)	2	0.48
	Non-Responders (Day 2)	7 (129)	13 (8)	0.04 (0.05)	11	0.38
48h	Baseline (Day 1)	14 (280)	29 (13)	0.05 (0.04)	44	0.05
	Responders (Day 2)	7 (60)	47 (19)	0.30 (0.08)	27	0.01
	Non-Responders (Day 2)	8 (155)	24 (13)	0.06 (0.04)	19	0.10

^1^5–12 day old, F_0_ females sugar starved 24h

^2^SAI = Spatial Activity Index

^3^SR = Signed rank test statistic

^4^Probability that SAI value is equal to zero

### Heritability of SR behaviors

**Fig 3:**
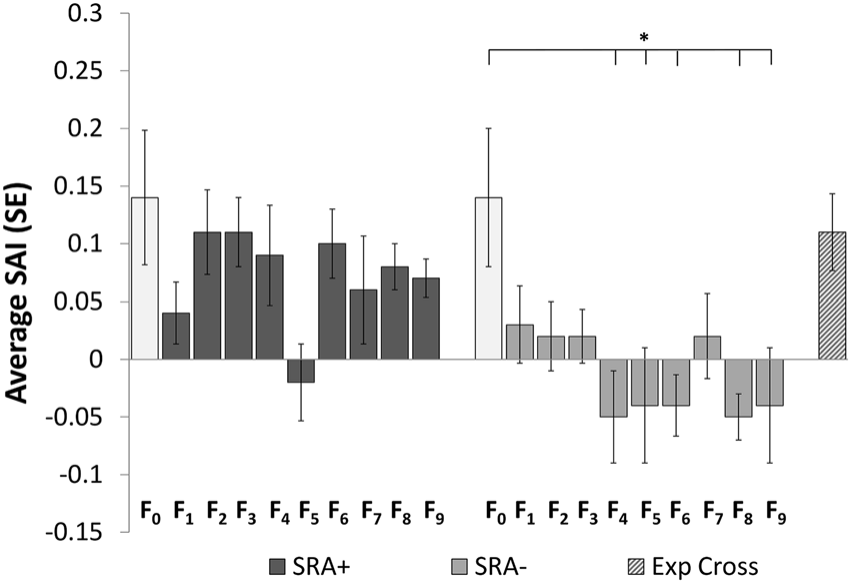
The heritability of spatial repellent insensitivity. Spatial activity index (SAI) values by generation in selectively bred *Ae*. *aegypti* responder (SRA+) and non-responder (SRA-) strains. * = SAI values significantly different from the baseline F_1_ generation via ANOVA with Dunnett’s test for multiple comparisons, α = 0.05. Exp Cross = F_9_ SRA- females mates with F_1_ wt males.

The baseline average SAI value for F_0_ female mosquitoes, which gave rise to all subsequent SRA^+^ and SRA^-^ lineages, was 0.14 ±0.06 (significantly greater than zero at *P*<0.02), confirming that parental mosquitoes were actively repelled by volatile transfluthrin in the assay system. Selective breeding experiments were then carried out through the F_9_ generation (Table 2 and Fig 3). SAI results from the unselected control strain (S4 Fig) and the SRA^+^ strain (Fig 3) did not indicate any changes in behavioral responses to volatile transfluthrin at any time point compared to baseline (no significant differences at *P* = 0.05). Results from the SRA^-^ strain, on the other hand, showed a steady decrease in SAI scores, which reached statistical significance (*P*<0.05) by the F_4_ generation (SAI = -0.05 ±0.04) (Fig 3). This SR insensitive phenotype was confirmed in each subsequent SRA^-^ generation, with the exception of the F_7_ cohort in which the reduced SAI value (0.02 ±0.03) was not significantly different from baseline at *P* = 0.05 (Fig 3).

**Table 2:** Spatial repellency behaviors in selectively bred Aedes aegypti^1^ responders (SRA+) and non-responders (SRA-).

	Number Trials (No. Mosqu.)	Mean Percent Active (SEM)	Mean SAI^2^ (SEM)	SR^3^	*p* ^*4*^	Cohort	Number Trials (No. Mosqu.)	Mean Percent Active (SEM)	Mean SAI^2^ (SEM)	SR^3^	*p* ^*4*^
*F*_*0*_	9 (180)	29 (27)	0.14 (0.06)	21	0.02	*F*_*0*_	9 (180)	29 (27)	0.14 (0.06)	21	0.02
*SRA* ^*+*^ *F*_*1*_	9 (180)	19 (14)	0.04 (0.03)	23	0.10	*SRA* ^*-*^ *F*_*1*_	9 (180)	26 (18)	0.03 (0.03)	16	0.11
*SRA* ^*+*^ *F*_*2*_	9 (180)	23 (11)	0.11 (0.04)	37	0.01	*SRA* ^*-*^ *F*_*2*_	9 (180)	9 (8)	0.02 (0.03)	6	0.34
*SRA* ^*+*^ *F*_*3*_	9 (180)	23 (14)	0.11 (0.03)	33	0.02	*SRA* ^*-*^ *F*_*3*_	9 (180)	10 (6)	0.02 (0.03)	11	0.29
*SRA* ^*+*^ *F*_*4*_	9 (180)	51 (13)	0.09 (0.04)	33	0.03	*SRA* ^*-*^ *F*_*4*_	9 (180)	36 (18)	-0.05 (0.04)	-20	0.13
*SRA* ^*+*^ *F*_*5*_	9 (180)	14 (12)	-0.02 (0.03)	-5	0.37	*SRA* ^*-*^ *F*_*5*_	9 (180)	22 (16)	-0.04 (0.05)	-8	0.32
*SRA* ^*+*^ *F*_*6*_	9 (180)	27 (11)	0.10 (0.03)	39	0.01	*SRA* ^*-*^ *F*_*6*_	9 (180)	25 (12)	-0.04 (0.03)	-21	0.10
*SRA* ^*+*^ *F*_*7*_	9 (180)	25 (11)	0.06 (0.05)	16	0.18	*SRA* ^*-*^ *F*_*7*_	9 (180)	34 (18)	0.02 (0.03)	7	0.29
*SRA* ^*+*^ *F*_*8*_	9 (180)	24 (17)	0.08 (0.02)	33	0.01	*SRA* ^*-*^ *F*_*8*_	9 (180)	28 (6)	-0.05 (0.02)	-33	0.10
*SRA* ^*+*^ *F*_*9*_	9 (180)	26 (10)	0.07 (0.02)	17	0.05	*SRA* ^*-*^ *F*_*9*_	9 (180)	31 (14)	-0.04 (0.05)	-13	0.25
						*Experimental Cross* ^*5*^	9 (180)	30 (15)	0.11 (0.03)	27	0.02

^1^5–12 day old females, sugar starved 24h

^2^SAI = Spatial Activity Index

^3^SR = Signed rank test statistic

^4^Probability that SAI value is equal to zero

^5^Experimental cross between F9 SRA- females and F1 wt (unselected) males

### Link between repellent insensitive and insecticide resistant phenotypes

**Fig 4:**
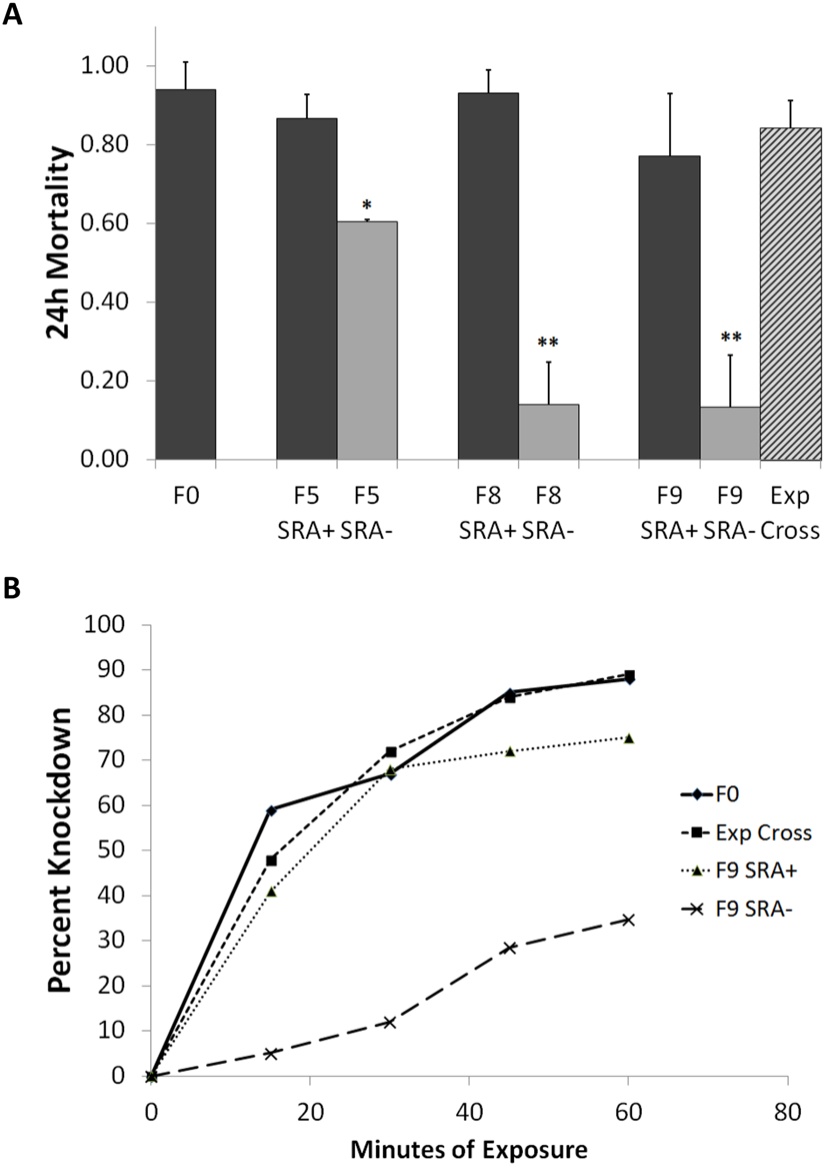
CDC bottle assay insecticide susceptibility patterns in selectively bred mosquito strains. (A) 24h mortality rates across various strains, asterisks signify significant differences from the baseline mortality rate * = *P*<0.05, ** = *P*<0.01. (B) Time to knockdown in a control (F1 unselected) strain and the F_9_ generation of SR responders (SRA+) and non-responders (SRA-) and the experimental cross (F_8_ SRA- females X F_1_ wt males).

Baseline CDC bottle tests indicated greater than 95% susceptibility to transfluthrin toxicity (24hr mortality) at the discriminating dose in the F_0_ parental mosquitoes that gave rise to all selectively bred strains (Fig 4). Insecticide susceptibility was then reevaluated in the F_5_ and F_8_ generations of colony and selectively bred mosquitoes (Fig 4). For the colony (unselected control, S5 Fig) and SRA^+^ (responder, Fig 4) strains, no significant changes in insecticide susceptibility were noted by either time to knockdown or 24hr mortality. In the selectively bred SRA^-^ repellent insensitive strain there was a moderate but significant (*P*<0.05) 23% reduction in mortality observed in the F_6_ generation compared to the control strain (60% ±1% vs. 95% ±6%) while the F_8_ SRA^-^ test population was highly resistant with a mortality of just 14% ±11%, a significant (*P*<0.01) 77% reduction in mortality compared to the unselected control (Fig 4A).

### Experimental cross of F_8_ SRA^-^ females and wild type F_0_ males restored both SR sensitivity and insecticide susceptibility

An additional round of selective breeding of F_8_ SRA^-^ non-responders gave rise to F_9_ SRA^-^ mosquitoes that continued to exhibit repellent insensitivity (SAI = -0.04 ±0.05) (Fig 3) as well as significantly decreased CDC bottle assay knockdown and 24h mortality (13% ±13%) (Fig 4). Mating females from the F_8_ SRA^-^ population with wild type F_0_ males newly colonized from the same location in Belize, however, restored both transfluthrin SR sensitivity (SAI = 0.11 ±0.03)(Table 2 and Fig 3) and insecticide susceptibility (24h mortality = 84% ±7%) in the resulting progeny (Fig 4).

### Differences in *kdr* allele frequency across SRA^-^ and SRA^+^ strains

**Fig 5:**
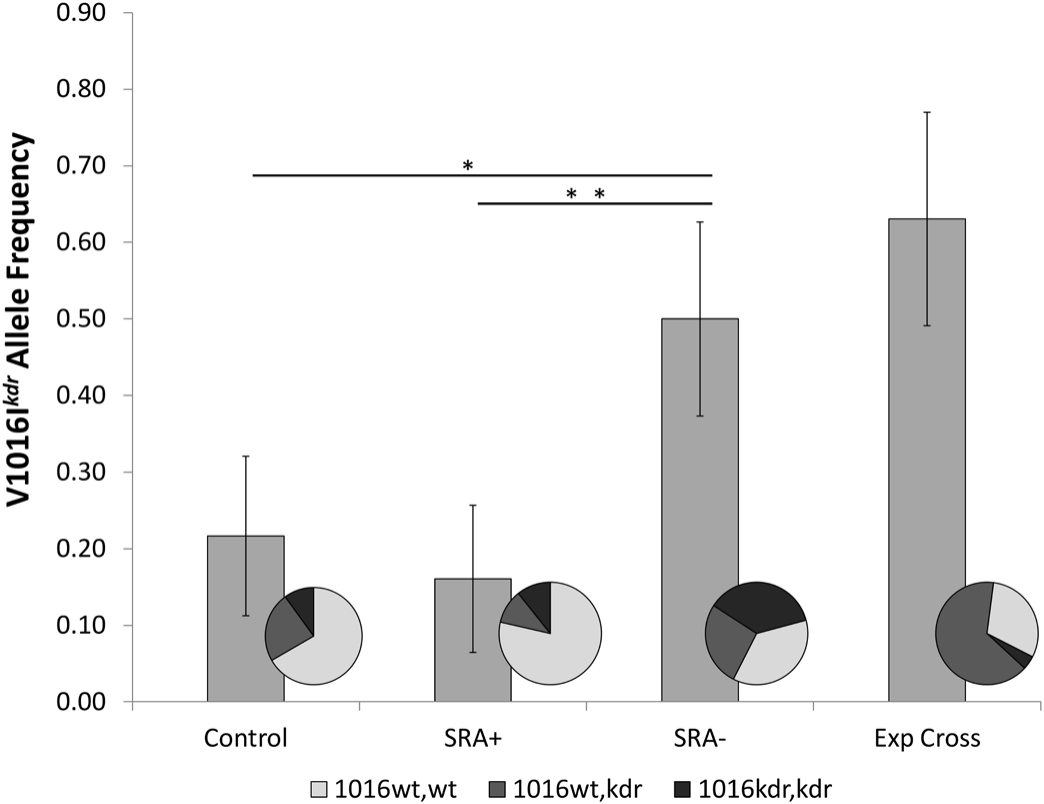
V1016I^*kdr*^ allele frequencies in F9 control, SRA+, SRA-, and experimental cross progeny cohorts. Bars indicate overall V1016I^*kdr*^ allele frequencies in samples of 30 mosquitoes from each mosquito strain. * = significant difference, *P<0*.*01*. Inlaid pie charts indicate the proportions of each cohort that were *wt* homozygous, *wt/kdr* heterozygous and homozygous *kdr* at position 1016.

Analysis of *kdr* allele frequencies was performed in the F_9_ control, F_9_ SRA^+^, F_9_ SRA^-^, and experimental cross progeny cohorts. Results indicated that the V1016I^*kdr*^ allele was more frequent (50%) in the SR insensitive, insecticide resistant SRA^-^ population than in the susceptible SRA^+^ (16%, *P*<0.01) or the control (22%, *P*<0.02) cohorts (Fig 5). Overall V1016I^*kdr*^ allele frequency remained high in the experimental cross progeny in which SR sensitivity and insecticide susceptibility were both restored (Fig 5). However, there was a significant (*P*<0.01) increase in the proportion of heterozygotes, from 27% in the SRA^-^ population to 65% in the experimental cross offspring (Fig 5). The assumption of Hardy-Weinberg equilibrium was rejected in both of the SRA+ (χ^2^ = 10.25, *P*<0.01) and SRA- strains (χ^2^ = 6.53, *P*<0.02), but not in either the control population or experimental cross progeny. There were no differences or changes in F1534C^*kdr*^ allele frequencies observed, with *kdr* prevalence over 90% for all cohorts tested.

## Discussion

The *in vitro* SR behaviors observed here were relatively plastic in that individual behavioral responses observed on day one were not consistent with subsequent behaviors observed upon identical chemical exposures at a later time point, reinforcing the notion that spatial repellency is a complex behavior with multiple determinants some of which are likely non-heritable [18]. Despite the overall high degree of variability in repellent behaviors on subsequent days, active SR responses were clearly more reproducible in mosquitoes that were given 48hr rest compared to those given only 24hr rest (Fig 4). This observation is consistent with other field [12] and laboratory [48] experiments that have shown post exposure habituation of mosquito behaviors that gradually resolves after appropriate recovery periods. The specific mechanisms driving these prolonged changes in behavior and their recovery, however, remain untested and in need of further investigation.

In the second set of experiments, SR responders (SRA^+^) and non-responders (SRA^-^) were identified and selectively bred for 9 generations. One of the possible outcomes of these experiments was the establishment of an SRA^+^ strain of *Ae*. *aegypti* with increased sensitivity to the SR action of volatile transfluthrin, and it was originally hypothesized that such a strain of super-responders might possess olfactory receptors with a particular affinity for detecting airborne transfluthrin. However, SR responses were not augmented in the selectively bred SRA^+^ strain at any time point. Conversely, there was a clear reduction in SR behaviors noted in the SRA^-^ strain, ultimately leading to a population of mosquitoes insensitive to the SR activity of volatile transfluthrin. These results do not preclude the possibility that transfluthrin might elicit some SR behaviors by activating and/or interrupting certain olfactory pathways. In fact, the reduction in repellent sensitivity observed in the SRA^-^ strain is in line with previous work by Stanczyk et al. (2010) that similarly demonstrated heritability of a DEET insensitivity trait in mosquitoes and further linked the phenomenon to changes in antennal olfactory reception [19]. Though similar in outcome, the DEET insensitivity trait described by Stanczyk et al. (2010) was clearly dominant, while the transfluthrin insensitivity observed here was restored after a single cross of SRA^-^ females with repellent sensitive wild type males. Additionally, the HITSS SR system used here is unique in that it is designed to permit the observation of directional mosquito movement absent any attractive stimuli, thus allowing for the measurement of active spatial repellency as a distinct entity not confounded by attraction inhibition. Accordingly, it is likely that the transfluthrin insensitive phenotype observed here relies on a different mechanism of action than the DEET insensitive phenotypes, which have been previously linked to changes in antennae sesillum function [26, 19].

As mentioned above, many insecticidal compounds are known to induce irritant and/or hyperactive responses in mosquitoes at sub-lethal doses [33, 12, 34], and this hyperactivity has been observed to promote the avoidance of treated surfaces [35]. These behavior modifying effects are sometimes referred to as excito-repellency, which is defined as the action of irritating a mosquito sufficiently so that it flies away from the source of the chemical before knockdown or death occurs [6, 23]. In this context, the strong correlation between reduced insecticide susceptibility in CDC bottle bioassays and SR insensitivity in HITSS bioassays observed in the selectively bred SRA^-^ strain suggests that the SR behaviors observed here resulted from neurotoxic irritation of mosquitoes by sub-lethal doses of airborne transfluthrin. This view is bolstered by the observed link between the SRA^-^ phenotype and an increase in the frequency of at least one target site mutation, the V1016I^*kdr*^ allele, which echoes previous reports of an association between *kdr* mutations and decreased excito-repellency behaviors in some field populations of *Anopheles* spp. exposed to pyrethroids [15, 49]. One weakness of the present study is that *kdr* allele frequencies were not established in the P_1_ parental population. However, the presence of the V1016I ^*kdr*^ allele in the F_9_ control (freely mating) population at low but stable frequencies does indicate that the allele was likely present in the parental strain and may have contributed to the less than 100% mortality observed in the baseline CDC bottle bioassays and was likely selected for during these experiments.

In addition to suggesting the neuro-physiological irritation of mosquitoes by active ingredient vapors as a primary mechanism by which transfluthrin can elicit SR behaviors in *Ae*. *aegypti*, the results of these selective breeding experiments are also notable for having experimentally reduced insecticide susceptibility in a population of vectors exposed only to sub-lethal doses of an airborne insecticide. This is of particular importance as one of the proposed benefits to the expanded use of spatial repellents in vector control programs is the potential to alleviate much of the selective pressure that encourages the emergence of insecticide resistance from sustained use of toxic interventions in the current vector control paradigm [7, 5, 8, 50]. Our results indicate that if a repellent elicits SR behaviors in the target vector through, at least in part, the same mechanisms that produce toxicity at higher doses, then the potential for selecting resistance traits might remain. Our observation that a single cross of SRA- females with wild type F_0_ males restored both SR sensitivity and insecticide susceptibility to offspring suggests that the insecticide resistant/SR insensitive phenotypes observed here were predominantly in V1016I^kdr^ homozygotes. This could indicate dominance of wild type voltage gated ion channel function over V1016^kdr^, and is predictable given that *kdr* mutations have been associated with high fitness costs in *Ae*. *aegypti* [51, 52]. It should be noted, however, that at least one other *kdr* allele, F1534C, was present at high frequencies (>90%) throughout this study, suggesting that any single allele represents only one factor contributing to the overall insecticide susceptibility and SR sensitivity profile of an individual mosquito. The relative contributions of various resistance traits, including metabolic mechanisms, to repellent insensitivity and to overall fitness need to be further elucidated.

It is also important to consider that when populations of SR responders and non-responders were allowed to mate freely (control strains), repellent sensitivity and insecticide susceptibility were maintained. The *in vitro* selective breeding approach used here favored the emergence of repellent insensitivity/decreased insecticide susceptibility only when SR insensitive females were mated exclusively with SR insensitive males. The degree to which natural mosquito populations would experience the same selective pressure in a standalone SR-based system is uncertain. Firstly, it is difficult to imagine a scenario in which repellent insensitive or repellent sensitive individuals that survive exposure to a volatile insecticide would significantly out-compete one another post-exposure, particularly when it has been shown that the use of coils to deliver airborne pyrethroids results in the decreased fitness of all mosquitoes, even those not repelled [7]. Additionally, it is not known how or to what degree chemical exposure to repellents might affect natural male mosquito populations in an operational setting, exposures that are likely to vary significantly according to where the active ingredient source is placed and the typical mating behaviors of the target vector.

Nonetheless, it is essential to consider these results while recognizing that pyrethroids are the most commonly used class of insecticide worldwide [5, 53, 54]. Indeed, for public health applications pyrethroid use constitutes the front line approach for both indoor residual spraying [55] and insecticide treated bed nets [56], resulting in significant and growing concerns over the rapid spread of pyrethroid resistance [39, 57, 38]. Against this backdrop, these findings are potentially more worrisome, as the effects of introducing a volatile pyrethroid repellent in an area where residual pyrethroids are already in use are unknown and require further evaluation and monitoring. As with insecticide resistance in general, the operational relevance of these findings are not known at this time. Clearly, more work must be done to define what these observations mean within the larger landscape of pyrethroid use, including how prolonged exposure to sub-lethal doses of volatile transfluthrin might impact insecticide resistance in natural vector populations and how already resistant populations might respond to a given repellent in the field. Furthermore, given the clear evidence that SR effects can produce beneficial public health outcomes [5–8, 13], these results suggest that an ideal SR compound would not only have a low toxic profile but also be unrelated to the chemical classes currently used in vector control. Acknowledging this highlights the pressing need to identify new insect behavior modifying compounds with novel mechanisms of action [58].

### Conclusions

Collectively, these results show that the *in vitro* SR responses observed here are complex behaviors with a mix of heritable and non-heritable determinants. Based on the link between the SR insensitive phenotype and decreased insecticide susceptibility, evidence also supports a model whereby sub-lethal doses of volatile transfluthrin can elicit SR responses in *Ae*. *aegypti* by inducing a hyperactive or agitated state via neurotoxic pathways, likely independent of olfactory stimulation or interruption. Care should be taken before extrapolating these results to other active ingredients or vector species. It should also be emphasized that these results do not indicate that transfluthrin elicits SR behaviors in *Ae*. *aegypti* exclusively by disrupting motor-neuron activity: olfactory and/or gustatory pathways may also play a role, whether via active detection and avoidance of odor cues or through the disruption of host detection and/or feeding, possibilities that should continue to be investigated using a variety of methods. Additionally, the appearance of decreased insecticide susceptibility and increased *kdr* allele frequency in the selectively bred offspring of mosquitoes exposed only to sub-lethal insecticide vapors raises some important questions about how the long-term use of repellents might impact vector populations over time. The answers to these questions will be dependent on several factors including which molecular mechanisms are driving specific repellent behaviors, the hereditary nature of repellent sensitivity and insensitivity, and other physiological effects of using sub-lethal concentrations of compounds that have insecticidal, as well as repellent, properties. Though the story is complex and further research is needed to better understand all of the physiological drivers of SR behaviors, evidence still supports the expanded use of spatial repellents in public health applications to control disease vectors, albeit with continued monitoring of potential changes in target vector repellent sensitivities and/or insecticide susceptibilities and a renewed emphasis on the need to develop new active ingredients with novel, non-toxic mechanisms of action.

## Supporting Information

S1 Table Establishment of a diagnostic dose of transfluthrin for use in CDC bottle bioassays. (XLSX)

S1 Fig *Aedes aegypti* (Belize) F_0_ insecticide susceptibilities. Baseline CDC bottle assay knockdown by time. Doses were: 75 μg/bottle DDT, 50 μg/bottle malathion, and 7.5 μg/bottle transfluthrin. 24 hr mortality was greater than 95% for all chemicals tested. For DDT and malathion, these are the standard CDC bottle assay diagnostic doses (Brogdon and Chan, 2013). For transfluthrin, the dosage corresponds to 50% of the recommended standard for permethrin (CDC bottle assay standards have yet to be established for transfluthrin).
(PDF)

S2 Fig Spatial repellency dose-response curve. Weighted spatial activity index (SAI) scores and non-contact mortality for unselected (control) female *Aedes aegypti* exposed to varying doses of volatile transfluthrin in the spatial repellency bioassay. Each concentration was tested with 9 replicates of 20 mosquitoes.* indicates an average SAI significantly different from zero at *P*<0.05, error bars indicate the standard error of the mean. Transfluthrin concentrations on the X-axis are shown relative to the standard field application rate (FAR), where 1xFAR = 1.35 mg/m^3^.
(PDF)

S3 Fig General experimental design. (A) The behavioral plasticity experiments, where individual mosquitoes were collected after an initial high throughput screening system (HITSS) spatial repellency assay (SRA) and re-tested on a subsequent day to estimate the consistency of the observed repellency behaviors. (B) The selective breeding experiments, where after each round of HITTS SRA testing, SRA^+^ males were selectively mated with SRA^+^ females and SRA- males were selectively mated with SRA- females(left), while a control strain was left untested and able to mate freely (right). Two experimental generations are illustrated.
(PDF)

S4 Fig The maintenance of spatial repellent sensitivity in the freely mating *Ae*. *aegypti* colony. Spatial activity index (SAI) values by generation in unselected (control) mosquitoes. There were no significant differences from the baseline in any generation (ANOVA with Dunnett’s test for multiple comparisons, α = 0.05).
(PDF)

S5 Fig CDC bottle assay insecticide susceptibility patterns in the control mosquito population. (A) 24h mortality rates (B) Time to knockdown.
(PDF)
