# Supplementary figures and images for: Insensitivity to the Spatial Repellent Action of Transfluthrin in Aedes aegypti: A Heritable Trait Associated with Decreased Insecticide Susceptibility
Source: PLoS Negl Trop Dis. 2015 Apr 16;9(4):e0003726. doi: 10.1371/journal.pntd.0003726 (PMC4400042; doi:10.1371/journal.pntd.0003726)

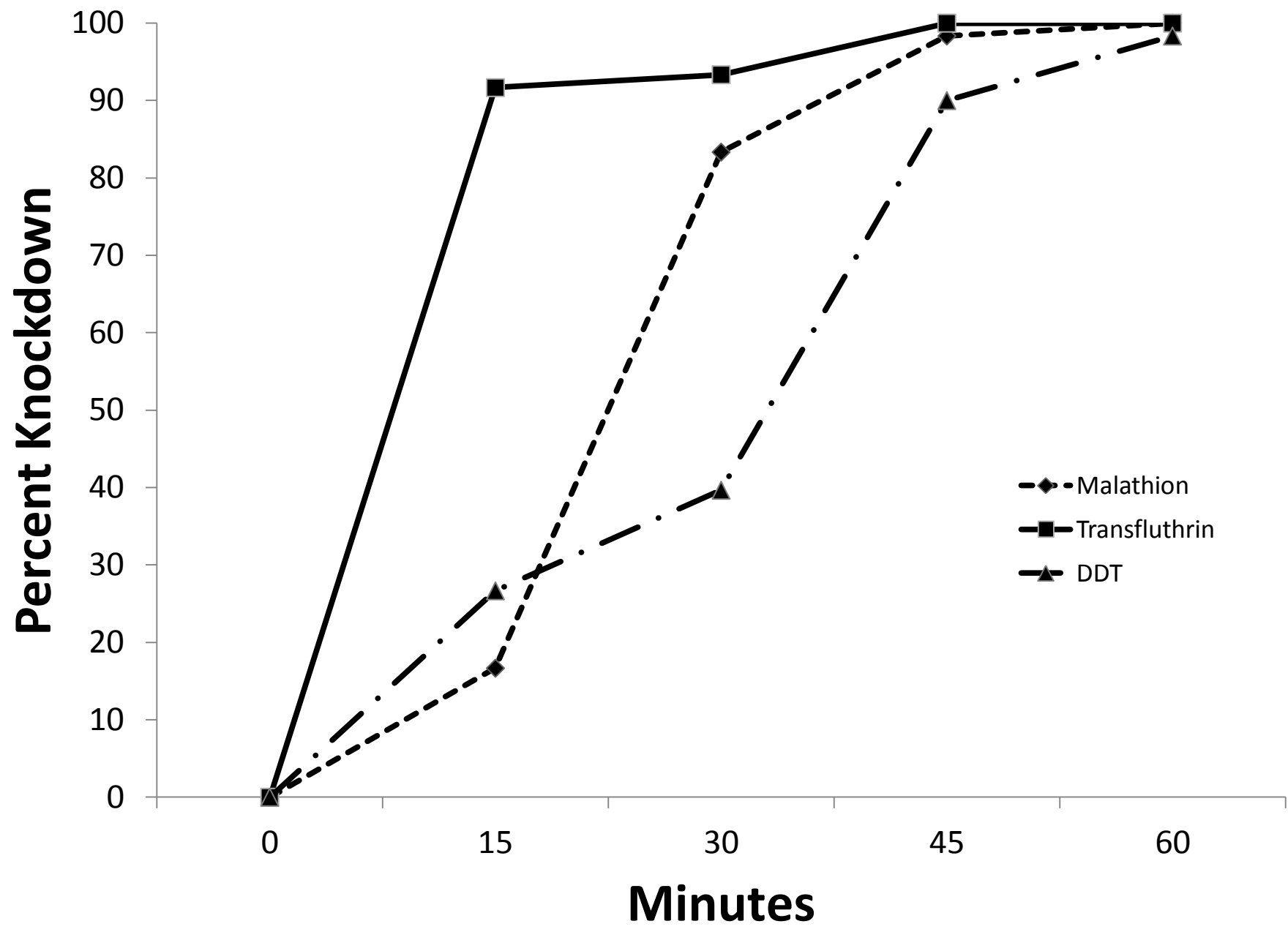

Supplement: S1 Fig — Baseline CDC bottle assay knockdown by time. Doses were: 75 μg/bottle DDT, 50 μg/bottle malathion, and 7.5 μg/bottle transfluthrin. 24 hr mortality was greater than 95% for all chemicals tested. For DDT and malathion, these are the standard CDC bottle assay diagnostic doses (Brogdon and Chan, 2013). For transfluthrin, the dosage corresponds to 50% of the recommended standard for permethrin (CDC bottle assay standards have yet to be established for transfluthrin). (PDF) [file pntd.0003726.s002.pdf]

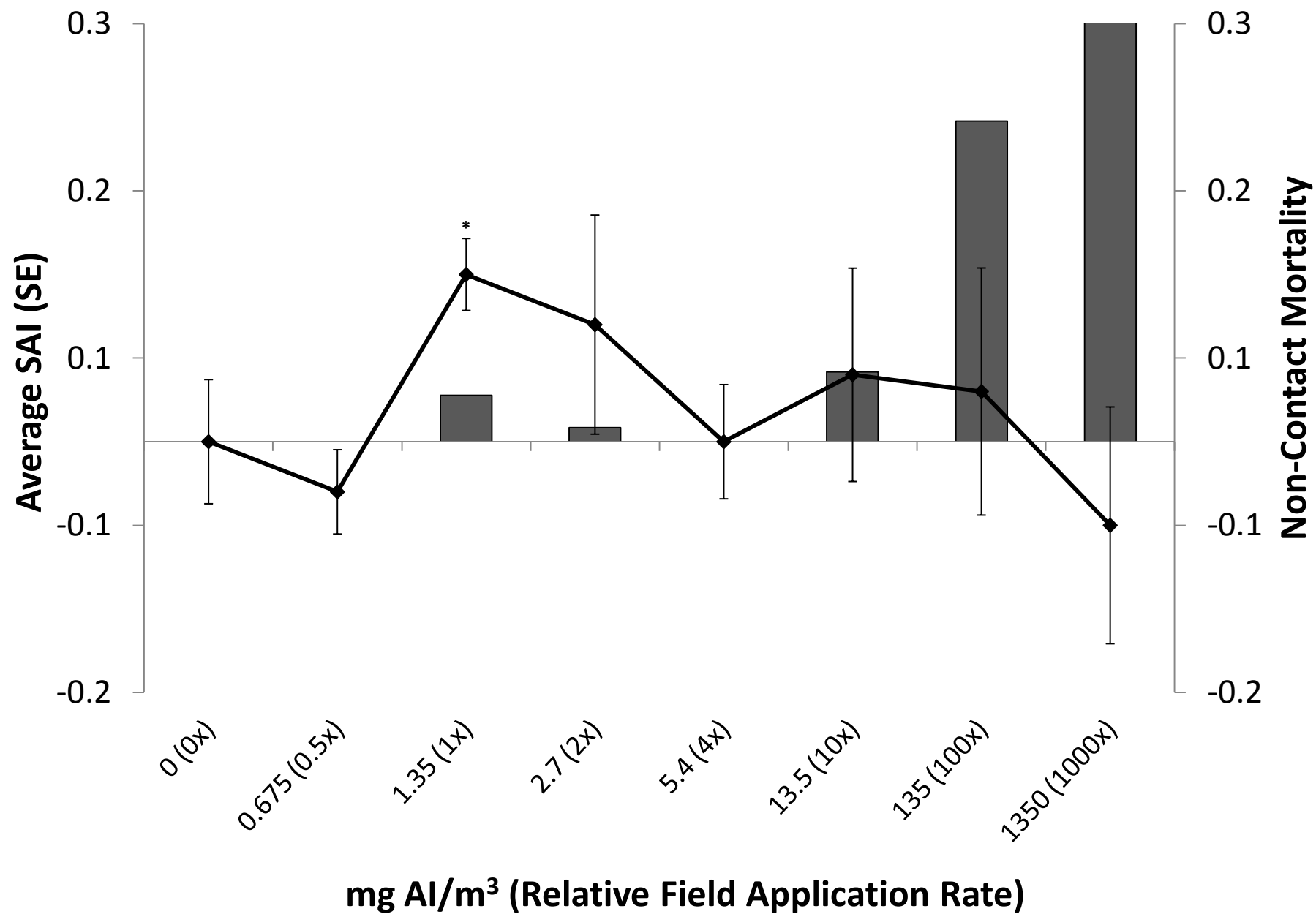

Supplement: S2 Fig — Weighted spatial activity index (SAI) scores and non-contact mortality for unselected (control) female Aedes aegypti exposed to varying doses of volatile transfluthrin in the spatial repellency bioassay. Each concentration was tested with 9 replicates of 20 mosquitoes.* indicates an average SAI significantly different from zero at P<0.05, error bars indicate the standard error of the mean. Transfluthrin concentrations on the X-axis are shown relative to the standard field application rate (FAR), where 1xFAR = 1.35 mg/m3. (PDF) [file pntd.0003726.s003.pdf]

**A**

Day 1

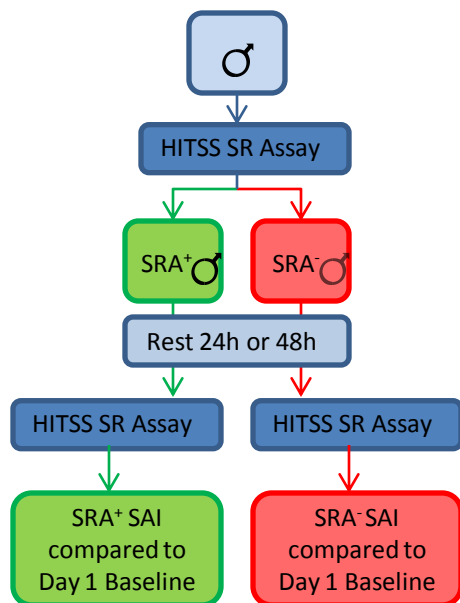

Day 2

**B** $F_0$  $F_1$  $F_2$ 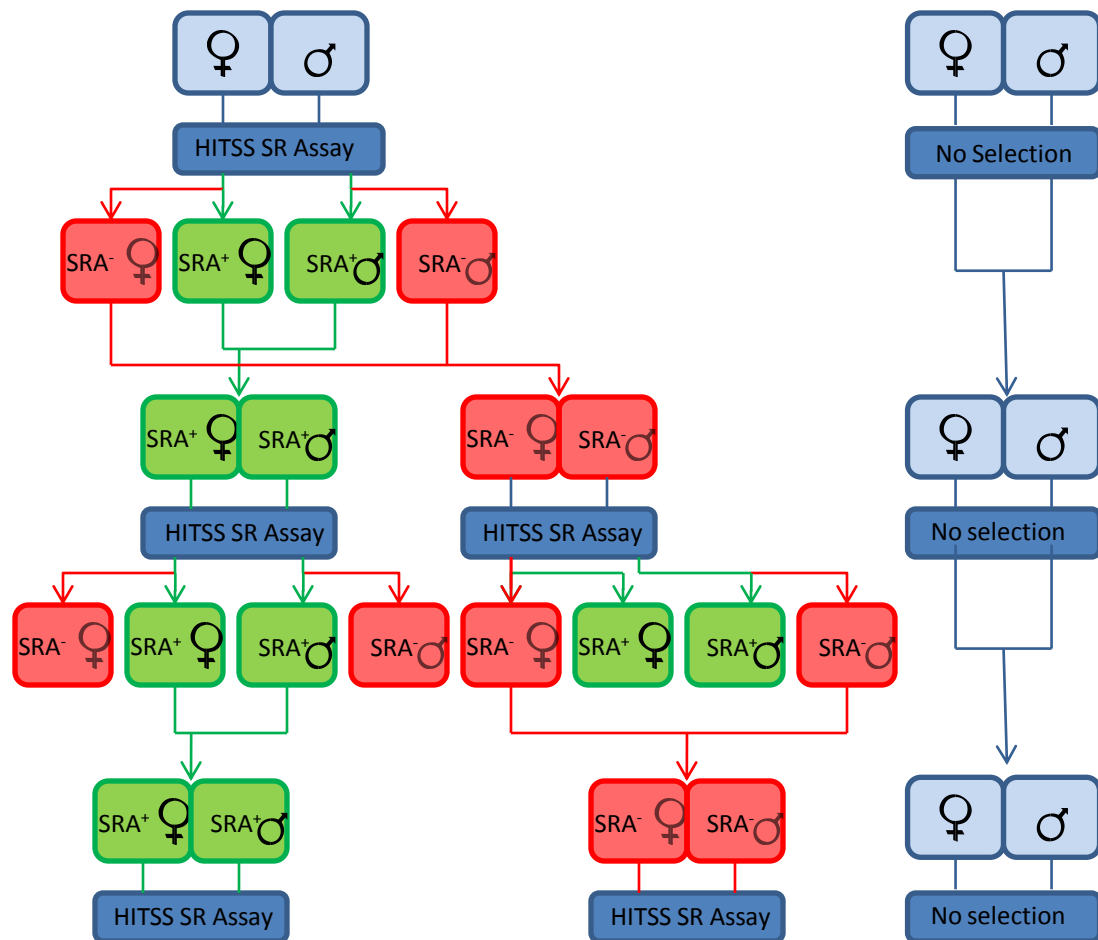

Supplement: S3 Fig — (A) The behavioral plasticity experiments, where individual mosquitoes were collected after an initial high throughput screening system (HITSS) spatial repellency assay (SRA) and re-tested on a subsequent day to estimate the consistency of the observed repellency behaviors. (B) The selective breeding experiments, where after each round of HITTS SRA testing, SRA+ males were selectively mated with SRA+ females and SRA- males were selectively mated with SRA- females(left), while a control strain was left untested and able to mate freely (right). Two experimental generations are illustrated. (PDF) [file pntd.0003726.s004.pdf]

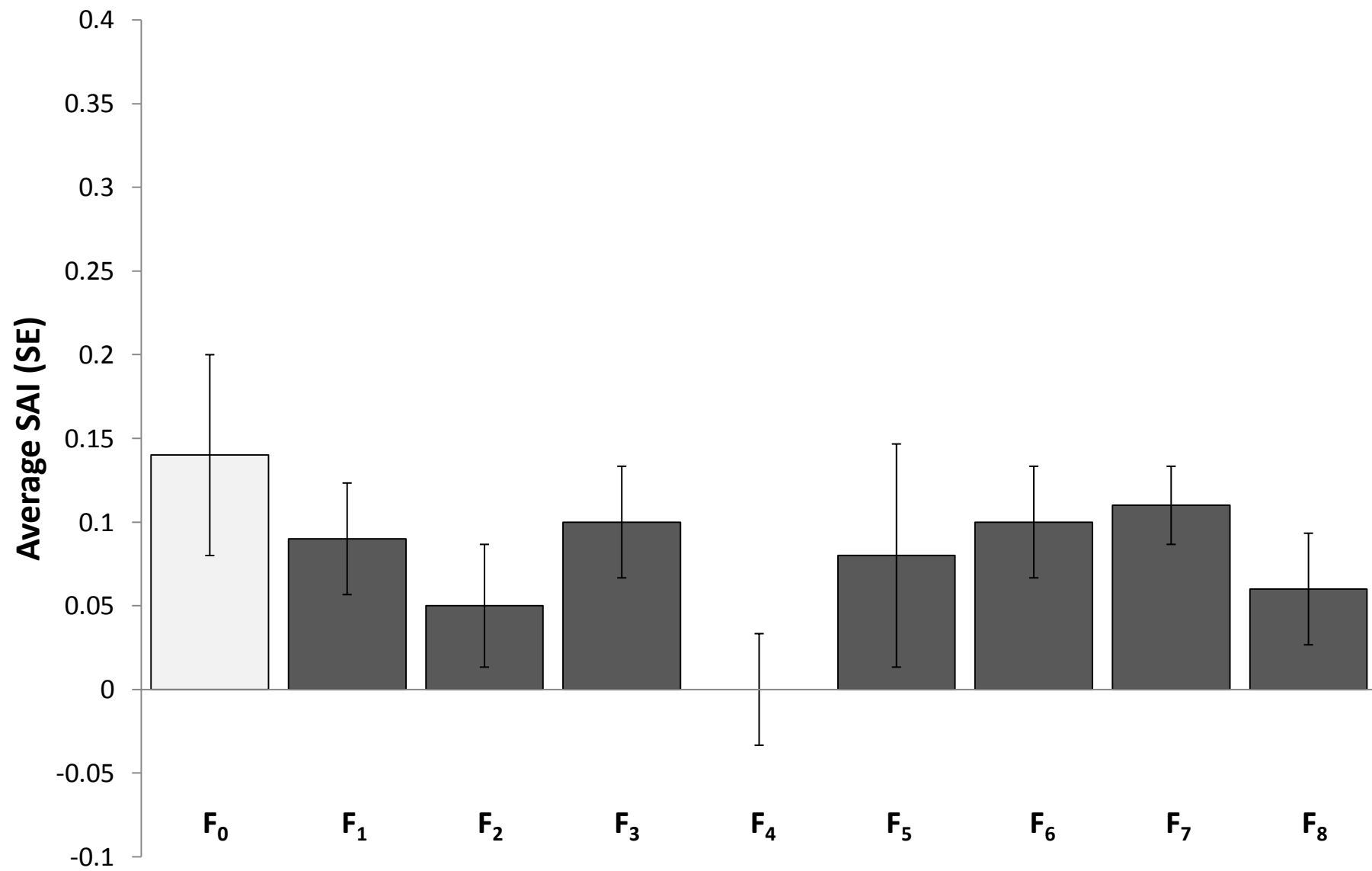

Supplement: S4 Fig — Spatial activity index (SAI) values by generation in unselected (control) mosquitoes. There were no significant differences from the baseline in any generation (ANOVA with Dunnett’s test for multiple comparisons, α = 0.05). (PDF) [file pntd.0003726.s005.pdf]

**A**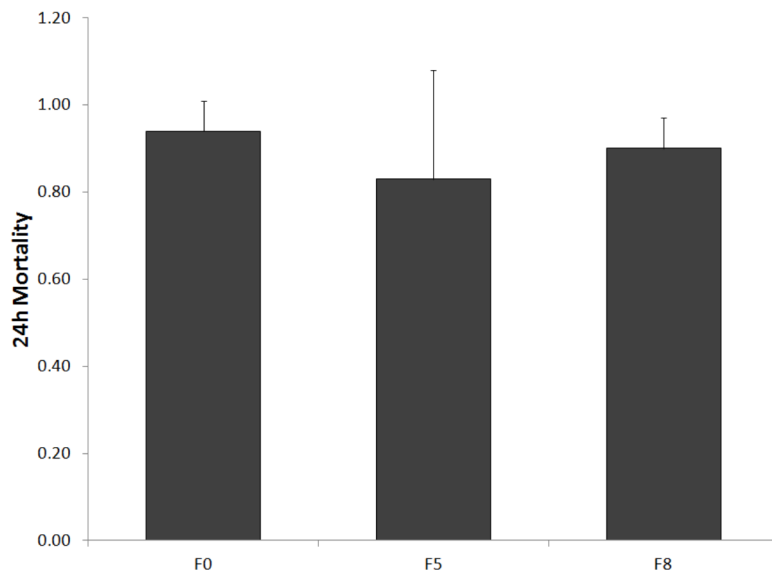**B**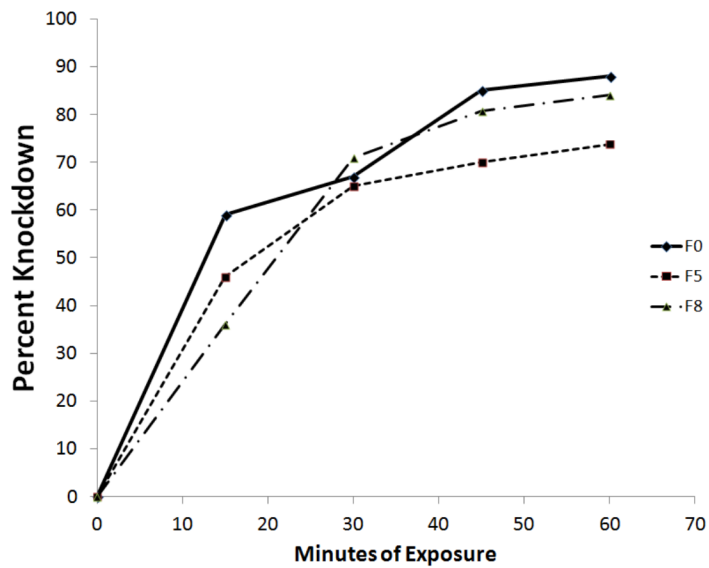

Supplement: S5 Fig — (A) 24h mortality rates (B) Time to knockdown. (PDF) [file pntd.0003726.s006.pdf]
